# Treatment of Acneiform Rash Due to Bromide of Potassium, While Continuing the Remedy

**Published:** 1882-02

**Authors:** 


### Treatment of Acneiform Rash due to Bromide of Potassium, while continuing the Remedy.

Duckworth reports the case of a woman, 20 years old, subject to epileptic attacks, and in whom sixty grains of bromide of potassium caused the appearance of a confluent acne which was very painful. The treatment consisted of the local application of the lotio sulphuris cum camphora; viz., ℞ Sulph. precip. ʒ ii, Spts. camphoræ ʒ i, Aquæ calcis ℥ ii, ♏ et ft. lot. Gruel was used instead of soap. There was improvement in a week, and in two weeks the hard and sluggish papules had disappeared; the bromide was taken as usual, with no return of the eruption. Mention is made of the fact of arsenic given together with the bromide controlling the eruption produced by the latter drug. Sangster, however, has found that a *sudden* increase in the dose of bromide has caused a rash, even though arsenic was being taken at the same time.—*St. Bartholomew's Hosp. Repts.*

